# Digital versus Traditional Workflow for Posterior Maxillary Rehabilitations Supported by One Straight and One Tilted Implant: A 3-Year Prospective Comparative Study

**DOI:** 10.1155/2018/4149107

**Published:** 2018-11-11

**Authors:** Francesco Ferrini, Paolo Capparé, Raffaele Vinci, Enrico F. Gherlone, Gianpaolo Sannino

**Affiliations:** Department of Dentistry, IRCCS San Raffaele Hospital and Dental School, Vita Salute University, Via Olgettina N. 48, Zip Code 20123 Milan, Italy

## Abstract

**Objectives:**

The aim of the study was to evaluate and compare digital and traditional prosthetic workflow for posterior maxillary restorations supported by an upright and a distally tilted implant at 3-year follow-up.

**Materials and Methods:**

Twenty-four patients were treated in the posterior maxilla with 24 immediately loaded axial and 24 distally tilted implants supporting 3-unit or 4-unit screw-retained prostheses. Three months after initial loading patients were randomly stratified into two groups: definitive traditional impressions were carried out in the control group, while digital impressions were performed in the test group. The framework-implant connection accuracy was evaluated by means intraoral digital radiographs at 3, 6, 12, and 36 months of follow-up examinations. Outcome considerations comprised implant and prosthetic survival and success rates, marginal bone level changes, and required clinical time to take impressions.

**Results:**

A total of 24 patients received immediately loaded screw-retained prostheses supported by an upright and a distally tilted implant (total 48 implants). No implant dropouts occurred, showing an overall survival rate of 100% for both groups. None of the 24 fixed prostheses were lost during the observation period (prosthetic survival rate of 100%). No statistically significant differences in marginal bone loss were found between control and test groups. The digital impression procedure required on average less clinical time than the conventional procedure.

**Conclusions:**

Clinical and radiologic results suggest that digital impression is a predictable procedure for posterior maxillary restorations supported by an upright and a distally tilted implant.

## 1. Introduction

Rehabilitation of the posterior maxilla represents a challenge for both patients and clinicians, due to bone resorption and maxillary sinus pneumatisation [1].

Different treatments have been proposed: short implants [2], crest augmentation [3], bone grafting and sinus elevation with crestal [4] or lateral approach [5], implants placed in pterygoid process [6], tuber [7], or zygoma [8]. However these procedures exhibit increased surgical and aesthetic risks as well as postsurgical morbidity. Furthermore costs related to such approaches often lead the patient towards cheaper removable prostheses, causing clinical and psychological drawbacks, especially in long-term edentulism.

Placing tilted implants has been suggested to overcome this therapeutic limitation and encouraging results have been obtained as reported by Chrcanovic et al. They concluded that implant angulation might not affect implant survival or marginal bone loss [9].

Agliardi et al. [10] demonstrated that immediate loading associated with tilted implants could be considered a viable treatment; the study was designed using a mesial axial implant and a distal tilted implant. This surgical technique could allow for the insertion of longer implants engaging three cortical layers, while coronal and apical part of implant might find anchorage in native bone with high level of primary stability.

However, surgical experience and Computed Tomography (CT) would be highly recommended for correct planning and surgical procedure [10, 11]. Moreover, implant placement and immediate loading should be delayed with a less than 4 mm residual bone height [5].

In the present study, in one group, implant position was recorded by digital impression [12, 13]. Indeed, dental impressions would be a crucial step in restorative dentistry. The intraoral situation is transferred to an extraoral cast, whose accuracy could influence the fit of the restorations affecting final restoration longevity [14].

Digital impressions have the potential to be faster and easier than conventional impressions, while reducing patient discomfort during prosthetic treatment and costs for the clinicians [12, 13].

Gimenez et al. tested in 2014 in a vitro study the accuracy of digital impression in tilted implants and demonstrated that angulated implants did not decrease the accuracy of the digital impression system [15].

Therefore, the aim of this study was to evaluate and compare the digital and the traditional workflows for posterior maxillary restorations supported by an upright and a distally tilted implant. The null hypothesis was that clinical and radiologic outcomes after 3 years of function with the definitive prostheses would be the same, regardless of whether digital or analogical workflow was adopted.

## 2. Materials and Methods

### 2.1. Patient Selection

This prospective study was performed at the Department of Dentistry, San Raffaele Hospital, Milan, Italy. Between February 2013 and March 2014, 24 patients (9=women, 15=men), aged between 41 and 72 years (mean age=63.6), were consecutively treated in the posterior maxilla with immediately loaded axial (24) and tilted (24) implants supporting 3-unit or 4-unit screw-retained prostheses.

The investigation was approved by the appropriate ethics committees related to the institution in which it was performed and was conducted according to the tenets of the Helsinki Declaration. STROBE (Strengthening the Reporting of Observational Studies in Epidemiology) guidelines (http://www.strobe-statement.org/) were followed.

The following exclusion criteria were adopted: presence of any active infection or severe inflammation in the areas intended for implant sites, presence of chronic systemic disease, any interfering medication such as steroid therapy or bisphosphonate therapy, radiation therapy to head or neck region within 5 years, smoking more than 15 cigarettes, bruxism habits, and poor oral hygiene. Diagnosis was made clinically and radiographically by panoramic radiograph and CT scan. Study casts were obtained from jaw impressions of the patients and mounted on articulators to fabricate temporary prostheses for immediate loading. Bone volume and quality were accurately assessed for a safe and prosthetically driven implant placement. All patients gave their written informed consent for immediate implant loading.

### 2.2. Surgical Procedure

One hour before surgery the patients received 2 g amoxicillin (Zimox, Pfizer Italia, Latina, Italy) and 1 g twice a day for a week after surgical procedure. Surgery was performed under anesthesia induced by local infiltrations of optocain solution with adrenaline 1:80.000 (AstraZeneca, Milan, Italy).

Incisions were made on the top of the alveolar crest, from the first molar to the first premolar region, and subperiosteal dissection was carried out on the palatal and vestibular surfaces.

The posterior implant was placed in second premolar position, close to and parallel to the anterior sinus wall. It was tilted distally approximately 30 to 40 degrees relative to the occlusal plane providing a first molar prosthetic emergence. The lower corner of the implant neck was positioned at bone level. Then the placement of the anterior axial implant was performed (6=canines, 20=first premolar). The posterior implants were 3.3 mm (4) and 3.8 mm (20) in diameter and 13 mm (18) or 15 mm (6) in length, while the anterior implants were either 3.3 mm (4) or 3.8 mm (20) in diameter and 13 mm (17) or 15 mm (7) in length (Winsix, Biosafin, Ancona, Italy). In soft bone underpreparation was performed to obtain high primary stability. All implants in immediate function had a final insertion torque of at least 35 Ncm. In all patients anterior implants were immediately positioned in postextraction sockets while only in 9 patients posterior implants were placed in healed sites. Straight (20) and angulated abutments (4) (17°, Extreme Abutment, EA® Winsix, Biosafin, Ancona, Italy) were used for anterior implants while 17° and 30° angulated abutments were screwed onto posterior implants to compensate for the lack of parallelism between implants as well as to place the prosthetic screw-access holes in an occlusal or lingual location. The angulated abutments were tightened with 25 N/cm of torque.

Flap adaptation and suturing were performed with 4-0 nonresorbable sutures (Vicryl; Ethicon, Johnson & Johnson, New Brunswick, NJ, USA). Nonsteroidal anti-inflammatory drugs (Brufen 600 mg, Abbott Laboratories, Chicago, IL, USA) and chlorhexidine digluconate 0.2% mouthwash during the first 2 weeks after surgery were prescribed as postoperative care for all participants.

### 2.3. Prosthetic Protocol

**Figure 1:**
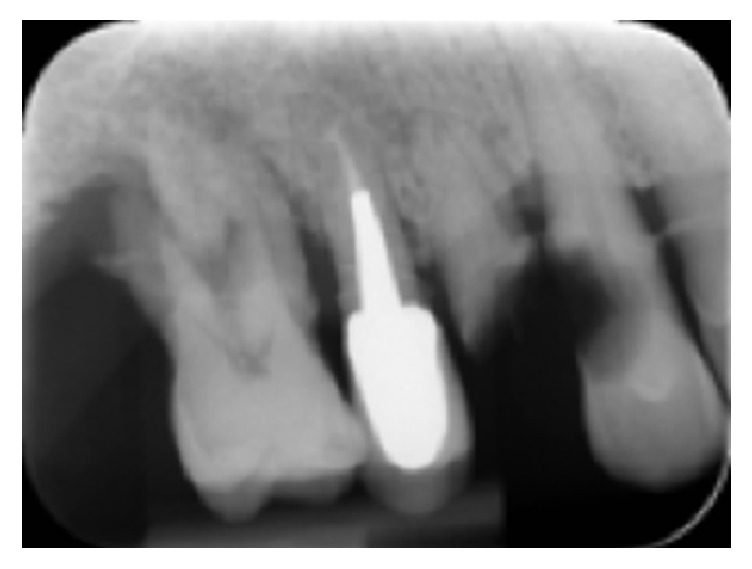
Presurgical radiographic view.

**Figure 2:**
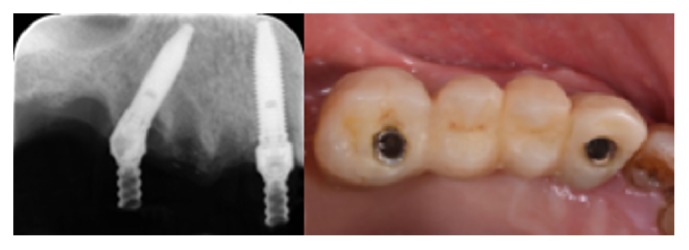
Prefabricated screw-retained, acrylic resin interim restorations were delivered immediately in all patients. The posterior implant was placed in second premolar position, distally tilted providing a first molar prosthetic emergence. The lack of parallelism between implants was compensated by means straight and angulated abutments.

Prefabricated screw-retained, acrylic resin interim restorations were delivered immediately in all patients (Figures 1 and 2). The interim prostheses were fabricated by a technician on the basis of the diagnostic wax-up and presented two openings according to planned abutment emergence. Straight cylinders (AT, Winsix, Biosafin, Ancona, Italy) were screwed onto the angulated abutments. The passive fitting and the occlusal relationship of the interim prostheses were checked and were then intraorally relined with autopolymerizing polyurethane resin (Voco, SC, USA). After polymerization the prostheses were removed from the implants and retention, stability, and marginal precision were improved by resin addition around the collar of the abutment. The screw-retained interim restorations were tightened with a 20 N/cm of torque.

Articulating paper (40 *μ*m Bausch Articulating Paper) was used to establish the presence of static occlusion, central contacts made on all masticatory units, or dynamic occlusion, including canine or premolar guidance. Occlusion was adjusted where necessary. Provisional resin (Fermit, Ivoclar Vivadent, Naturno) was used to cover screw access holes.

Patients were advised to adhere to a soft diet for the first 2 months after surgery and then to return to a regular diet but avoid harder food items for another 2 months.

After 3 months in function with the provisional, definitive restorative procedures were started.

Patients were randomly selected by lots in closed envelopes to be allocated in control or test group. The allocation was performed by a blind operator (PC).

**Figure 3:**
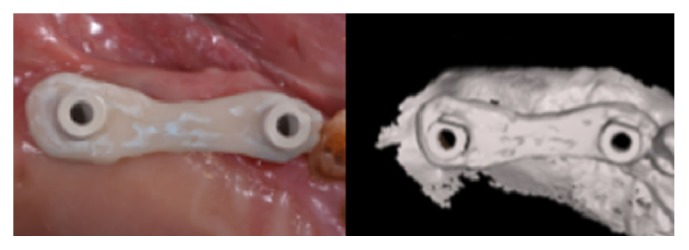
In the test group scan bodies were splinted as impression guide for a digital implant level impression.

**Figure 4:**
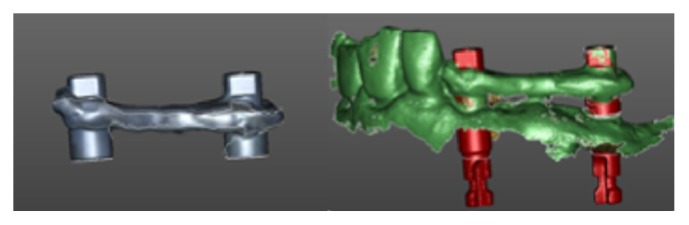
The impression guide was extraorally scanned by a laboratory scanner. A dental CAD software was then used to match the extraoral scanning to the intraoral impressions.

**Figure 5:**
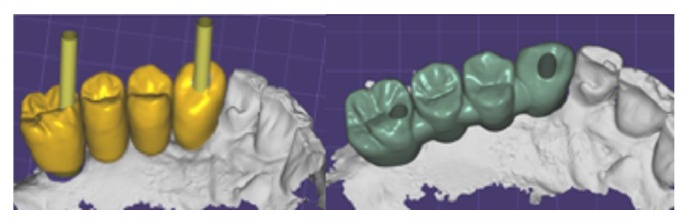
Digital planning. Prosthetic screw-access holes were placed in an occlusal or lingual location thanks to angulated abutments.

In the control group (CG=12), traditional pickup implant level impressions were taken (Permadyne, ESPE), while in the test group (TG=12) the scan bodies were used as impression copings for a digital implant level impression (3MTM True Definition Scanner). Orthodontic wire and resin were used to splint the scan bodies (Figure 3). After the intraoral scanning the impression guide was coated with a scanning powder and scanned by a laboratory scanner (Deluxe, OpenTechnologies, Brescia, Italy) as well. The new extraoral scanning of the impression guide, more accurate than intraoral one, was matched to the scan of the guide generated by the intraoral scanning thanks to a dental CAD software (Exocad GmbH, Germany) (Figure 4). The scan of the impression guide was used as a master guide during all the prosthetic phase (Figure 5).

**Figure 6:**
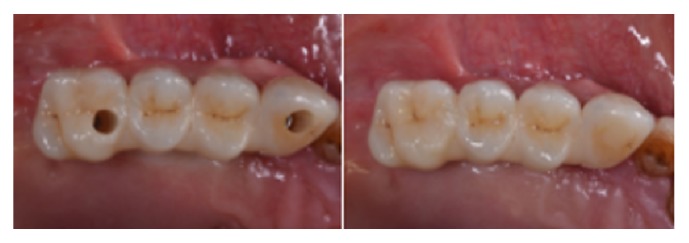
Occlusal view of definitive milled high-precision screw-retained zirconia-ceramic framework prosthesis screwed into the implants.

**Figure 7:**
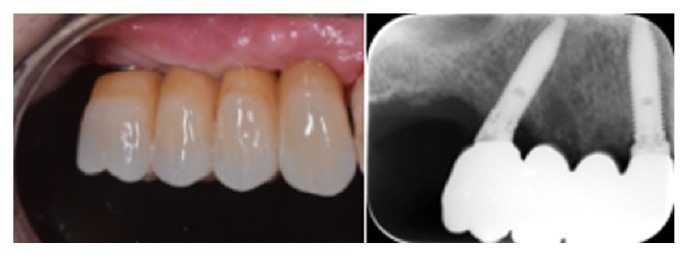
Vestibular and radiographic views of definitive restoration at 36-month follow-up.

Definitive milled high-precision screw-retained zirconia (Nacera Shell, Milan, Italy) anatomical framework prostheses were produced. Veneering was performed by ceramic (Initial Ceramics, GC, US) after screwing zirconia framework onto implants analogs, which were positioned in an epoxy resin (XM 24, 3M, MN, US) stereolithographic model (iPro 8000 MP Printer, 3D Systems) (Figures 6 and 7).

Outcomes of impression techniques were evaluated using the following clinical acceptance criteria: (1) accurate imprinting of implant areas; (2) no voids on the occlusal, buccal, and lingual sides; and (3) proper reproduction from the vestibule up to the mucogingival junction [12]. Impressions not meeting the criteria underwent retakes for conventional impressions or rescans/further scans.

Total treatment time and retakes/rescans required to meet acceptance criteria were evaluated to prove the efficacy of the two impression techniques. Treatment time (minutes/seconds) was represented by the time required to obtain an acceptable impression, in accordance with criteria (procedure time). When necessary, impression retakes (conventional impressions) and rescans of missing areas (digital impressions) were registered as extra working time and additional events

### 2.4. Follow-Up

Follow-up visits were scheduled at 3, 6, 12, 24, and 36 months after implant insertion.

Implant survival was defined by implant stability, absence of pain, mucosal suppuration, or radiolucent zones surrounding the implants at the time of examination. Implant success was defined as implant survival with marginal bone loss of less than 1.5 mm after 1 year of loading and no more than 0.2 mm of loss between each follow-up visit after the first year in function.

The efficiency of digital impression as well as restoration success was evaluated by accurate imprint of implant areas, absence of voids on the occlusal, buccal, and lingual sides, fit of the prostheses, and absence of fractures in the glass-ceramic veneered zirconia superstructure.

Biological and prosthetic complications (number and type) were recorded as single episodes for each implant. Particular attention was used to assess peri-implantitis (defined as progressive bone loss with sign of infections around an osseointegrated implant), presence of pain, presence of pus, paresthesia in the lower jaw, and implant fracture.

Intraoral digital radiographic exams (Schick CDR, Schick Technologies) were taken immediately after the insertion of the fixture, at 6, 12, and 36 months to verify the marginal precision of 24 definitive prosthetic frameworks fixed onto the implants. They were made perpendicular to the long axis of the implant with long-cone parallel technique, using an occlusal custom template to measure the marginal bone level. A dedicated dentist measured the changes in crestal bone height over time. The difference in bone level was measured radiographically through specific software (DIGORA 2.5, Soredex, Tuusula, Finland). The software was calibrated for every single image using the known implant diameter at the most coronal portion of the implant. The linear distance between most coronal point of bone-to-implant contact and the coronal margin of the implant collar was measured to the nearest 0.01 mm, at both mesial and distal sides, and averaged.

Bone level changes at single implants were averaged at group level.

### 2.5. Statistics

Statistical analysis was performed with the statistical software SPSS 14 for Windows (SPSS).

Descriptive analysis was performed using mean and standard deviation. Time needed for digital and conventional procedures was measured in seconds and reported as means ± standard deviations. Marginal bone loss around the upright and tilted implants was compared between groups by means of the Student t-test at a significance level of P = 0.05.

To compare TG and CG in terms of treatment time and number of retakes/rescans, the Wilcoxon signed-rank test was used.* P *values at < .05 were considered statistically significant.

## 3. Results

A total of 24 patients were treated in the posterior maxilla with immediately loaded 3-unit (n.6) or 4-unit (n.18) screw-retained prostheses supported by 1 axial (24) and 1 distally tilted (24) implants.

Twenty-four definitive milled high-precision screw-retained zirconia ceramic framework prostheses were fabricated. No implant dropout occurred. The mean (SD) implant length was 13.66 mm (±2.8 mm) for the axial and 13.5 mm (±1.96 mm) for the tilted implants in the control group, while the mean (SD) implant length was 13.5 mm (±2.8 mm) for the axial and 13.66 mm (±1.96 mm) for the tilted implants in the test group. The mean (SD) values of implant tilting were 33.89 degrees (±6.7) and 32.75 degrees (±6.9) in the control and test group, respectively.

The radiographic examination showed the prosthesis-implant connection accuracy. The implant survival rate was 100%, while none of the 24 prostheses were lost during the observation period (prosthetic survival rate 100%). Six occlusal screw loosening incidences of definitive prosthesis (n.4 CG, n.2 TG) were recorded at 6-month follow-up visit. No biological or mechanical complications (such as screw loosening and/or fracture, zirconia framework fracture, and chipping of the veneering material) occurred during the whole follow-up period.

The 3-year overall implant survival rate was 100% for axially positioned implants as well as for tilted implants in both groups.

Radiographic results are reported in Table 1. At the 36-month evaluation, peri-implant crestal bone loss averaged 1.09 ± 0.52 mm for upright implants (n = 12 implants) and 1.03 ± 0.65 mm for tilted implants (n = 12 implants) in the control group, while the mean marginal bone level at the 3-year follow-up was 1.10 ± 0.39 mm for upright implants (n = 12 implants) and 1.04 ± 0.42 mm for tilted implants (n = 12 implants) in the test group (Table 1).

No statistically significant differences (P>0.05) in crestal bone loss between tilted and upright implants were detected at 6, 12, and 36 months of follow-up evaluation.

The analysis of procedure time revealed that the digital impression procedure took less time than the conventional procedure (Table 2), and the difference was statistically significant (P < 0.05). No rescans for digital impressions or retakes for conventional impressions were required.

## 4. Discussion

The 3-year clinical and radiologic results of this prospective study have shown how the impression accuracy of both traditional and digital protocol positively affect the prognoses of posterior maxillary restorations supported by an upright and a distally tilted implant.

No implants were lost during the observation period. The implant (100%) and prosthesis (100%) survival rates compared favorably with results shown in previous studies, in which partial prostheses supported by axial and tilted implants were observed for up to 3 years after loading [10, 11].

Cumulative previously reported implant survival rates of axial and tilted implants have been similar [16, 17]. Krekmanov et al. [18] studied tilting of posterior implants for improved prosthesis support in 47 patients and found that there were no implant failures in mandible while cumulative success rates in the maxilla at five years were 98% for tilted implants and 93% for nontilted implants. Agliardi et al. [19] evaluated prognosis of immediately loaded fixed full prostheses for treatment of edentulous patients with extreme bone loss in posterior mandibular region over average period of 30.1 months and found excellent outcome.

Bone marginal level changes evaluated on the mesial and distal surfaces for tilted implants in both groups were similar to other studies [20, 21].

Calandriello et al. [22] demonstrated that bone loss around an implant placed at an angle is same or less as compared to that around axial implant.

In the present study, there was not statistically significant differences between groups.

Marginal bone loss was not affected by tilting the implants. Tilted implants were splinted to axial implants and exhibited a bone remodeling pattern similar to that reported in previous studies [17, 22].

In such protocol, bone anchorage and the rigid splinting provided by the interim prosthesis would be crucial to achieve a high primary implant stability and the following osseointegration process [22, 23].

Tilted implants were engaged in dense cortical bone structures, achieving tricortical anchorage while avoiding the need for bone grafting [22]. Longer implants were placed, and implant-to-bone contact area as well as primary implant stability was improved. According to previous clinical studies as well as numerical model results a better load distribution was achieved in the whole structure since prosthetic cantilever was eliminated [24–27].

A limited implant inclination (between 15 and 30 degrees) has been recommended by several authors [22, 27, 28].

In the present study, the mean (SD) implant tilting degrees were 33.89 degrees and 32.75 degrees in the control and test group, respectively, and had no deleterious effects on the load transfer to the surrounding bone. Moreover implant tilting did not decrease the accuracy of the digital impression system tested according to what was previously shown by Gimenez et al. [15].

Six occlusal screw loosening incidences of definitive restorations were recorded at 6-month follow-up visit probably due to occasional parafunctional habits or poor occlusal equilibration. No framework fractures or veneering chippings occurred during the whole follow-up period (prosthetic survival rate 100%).

No clinical and radiographic difference was found between the two groups of patients. However, in this study digital impressions showed greater efficacy than traditional impressions due to a shortened clinical time as well as an improved patient comfort. The risk for material distortion was eliminated and the 3D previsualization allowed for a real-time check of the scanning correctness. Moreover, the digital intraoral scanning combined with a CAD/CAM system allowed for a whole digital workflow. A physical dental model with repositionable implant analogs may be easily fabricated thanks to digital processing when needed.

Several clinical studies have been carried out to evaluate accuracy and precision of digital impressions versus traditional impressions.

An in vitro study compared Lava COS with an Impregum impression case. Accuracy was represented by “trueness” and “precision”, where the first was relative to the discrepancy between the model and the actual object size, whereas the latter referred to the fluctuation in the various measurements [29].

The digital system was able to produce improved trueness compared with the traditional impression. Concerning the marginal fit a recent clinical study reported that zirconia crowns fabricated from digital intraoral impressions with active wavefront sampling showed a lower median marginal gap (49 mm) compared to those derived by conventional impressions followed by the same CAD/CAM technology (71 mm) [30–32].

In a 3-year retrospective study [33] the clinical performance of glass-ceramic/zirconia crowns fabricated using intraoral digital impressions was evaluated confirming an impression accuracy fully comparable with conventional impression techniques. Furthermore, the digital workflow has been advocated to be almost threefold more efficient than the established conventional pathway for fixed implant-supported crowns [34].

## 5. Conclusions

Within the limitations of the present study, clinical and radiologic results suggest that the prosthetic digital workflow positively affects the prognoses of posterior maxillary restorations supported by an upright and a distally tilted implant. The digital scanning could be considered a reliable alternative to the traditional impression. The whole digital workflow may shorten clinical time and improve the patient acceptance. Further long-term prospective clinical trials are needed to confirm the effectiveness of digital impression procedure.

## Figures and Tables

**Table 1:** Crestal bone loss values (mean±SD) for tilted and upright implants (n=implant=48).

	Upright	Tilted
Bone Loss	maxilla n=24	maxilla n=24

6 months (mm)	0.98 ± 0.35	1.01 ± 0.40

12 months (mm)	1.04 ± 0.39	1.05 ± 0.48

36 months (mm)	1.09 ± 0.46	1.04 ± 0.54

**Table 2:** Analysis of procedure time for control group and test group.

Parameter	Conventional	Digital	P value
Procedure time (min:s)	06:41	03:32	<0.05

Additional time (min:s)	0	0	-

No. of retakes/rescans	0	0	-

## Data Availability

The data used to support the findings of this study are included within the article.
